# Identification of a novel bile marker clusterin and a public online prediction platform based on deep learning for cholangiocarcinoma

**DOI:** 10.1186/s12916-023-02990-9

**Published:** 2023-08-08

**Authors:** Long Gao, Yanyan Lin, Ping Yue, Shuyan Li, Yong Zhang, Ningning Mi, Mingzhen Bai, Wenkang Fu, Zhili Xia, Ningzu Jiang, Jie Cao, Man Yang, Yanni Ma, Fanxiang Zhang, Chao Zhang, Joseph W. Leung, Shun He, Jinqiu Yuan, Wenbo Meng, Xun Li

**Affiliations:** 1 The First School of Clinical Medicine, Lanzhou University, Lanzhou, 730030 Gansu China; 2 Department of General Surgery, The First Hospital of Lanzhou University, Lanzhou, 730030 Gansu China; 3 Gansu Province Key Laboratory of Biological Therapy and Regenerative Medicine Transformation, Lanzhou, 730030 Gansu China; 4 School of Medical Information and Engineering, Xuzhou Medical University, Xuzhou, 221004 Jiangsu China; 5 Clinical Research Center, Big Data Center, The Seventh Affiliated Hospital, Sun Yat-Sen University, Shenzhen, 518107 Guangdong China; 6 Division of Gastroenterology, UC Davis Medical Center and Sacramento VA Medical Center, Sacramento, CA 95817 USA; 7 Department of Endoscopy, National Cancer Center/National Clinical Research Center for Cancer/Cancer Hospital, Chinese Academy of Medical Sciences and Peking Union Medical College, Beijing, China

**Keywords:** Cholangiocarcinoma, Clusterin, Deep learning, Diagnostic biomarker, Proteomics

## Abstract

**Background:**

Cholangiocarcinoma (CCA) is a highly aggressive malignant tumor, and its diagnosis is still a challenge. This study aimed to identify a novel bile marker for CCA diagnosis based on proteomics and establish a diagnostic model with deep learning.

**Methods:**

A total of 644 subjects (236 CCA and 408 non-CCA) from two independent centers were divided into discovery, cross-validation, and external validation sets for the study. Candidate bile markers were identified by three proteomics data and validated on 635 clinical humoral specimens and 121 tissue specimens. A diagnostic multi-analyte model containing bile and serum biomarkers was established in cross-validation set by deep learning and validated in an independent external cohort.

**Results:**

The results of proteomics analysis and clinical specimen verification showed that bile clusterin (CLU) was significantly higher in CCA body fluids. Based on 376 subjects in the cross-validation set, ROC analysis indicated that bile CLU had a satisfactory diagnostic power (AUC: 0.852, sensitivity: 73.6%, specificity: 90.1%). Building on bile CLU and 63 serum markers, deep learning established a diagnostic model incorporating seven factors (CLU, CA19-9, IBIL, GGT, LDL-C, TG, and TBA), which showed a high diagnostic utility (AUC: 0.947, sensitivity: 90.3%, specificity: 84.9%). External validation in an independent cohort (*n* = 259) resulted in a similar accuracy for the detection of CCA. Finally, for the convenience of operation, a user-friendly prediction platform was built online for CCA.

**Conclusions:**

This is the largest and most comprehensive study combining bile and serum biomarkers to differentiate CCA. This diagnostic model may potentially be used to detect CCA.

**Supplementary Information:**

The online version contains supplementary material available at 10.1186/s12916-023-02990-9.

## Background

Cholangiocarcinoma (CCA) is known as a highly aggressive malignancy. According to the anatomical site of the lesion, CCA can be divided into intrahepatic cholangiocarcinoma (iCCA), perihilar cholangiocarcinoma (pCCA), and distal cholangiocarcinoma (dCCA). As the second most common malignant tumor in the hepatobiliary system, CCA accounts for about ~ 3% of all gastrointestinal tumors [1] and has a poor prognosis with low 5-year survival (7 to 20%) and high fatality rate (accounting for about 2% of the global annual cancer-related deaths), all of which boils down to its difficulty in early diagnosis [2, 3].

Diagnosing CCA is difficult because of its silent clinical character and anatomic location. Currently, CCA is mainly detected by imaging methods, such as computed tomography (CT), magnetic resonance imaging (MRI), and endoscopy, but their diagnostic powers are disappointing with a modest accuracy and an estimated sensitivity of only 6 to 71.9% [4, 5]. Serum CA19-9 is commonly used for CCA diagnosis, but its sensitivity and specificity are frustrating at best [6, 7]. Surprisingly, post-operative pathology results report that 10–25% of patients who underwent surgical management for suspected CCA are ultimately free of cancer cells, highlighting an urgent need for more accurate diagnostic tools [5, 8, 9].

Bile is the direct microenvironment for the growth of bile duct tumor cells, and cancer-related proteins in CCA can be secreted into the bile and may potentially be used as biomarkers for diagnosis [10, 11]. In addition, many serum markers also change in CCA [7, 12]. The differentially expressed proteins in the bile mainly reflect local changes while the serum markers mainly reflect systematic changes in CCA progression [4]. Thus, combining markers in the bile and blood could improve the accuracy in distinguishing CCA from other biliary diseases.

In this study, we identified and evaluated the power of a novel bile biomarker for the diagnosis of CCA. Based on this, a deep learning model was established by combining other serum markers. Finally, the diagnostic performance of the model was validated by another independent group.

## Methods

### Patient populations

This study was approved by the Human Research Ethics Committee of the First Hospital of Lanzhou University (LDYYLL2022-381) with an exemption of informed consent and was conducted in accordance with the Declaration of Helsinki principles. Clinical specimens came from two centers.

A total of 644 patients were divided into a discovery set, cross-validation set, and external validation set for the study (Additional file 1: Fig. S1). In the discovery set, the bile from 5 CCA patients and 4 patients with bile duct stones in the First Hospital of Lanzhou University was collected for proteomics analysis. In the cross-validation set, 376 patients (193 males and 183 females) were recruited from the Department of General Surgery of the First Hospital of Lanzhou University, including 144 CCA patients and 232 non-CCA patients from September 2018 to May 2022. The non-CCA group mainly consisted of benign biliary diseases and non-CCA cancers; benign biliary diseases included chronic biliary tract diseases and non-chronic biliary tract diseases. The chronic biliary tract diseases included bile duct stones (common bile duct (CBD) stones and intrahepatic bile duct (IBD) stones), cholangitic stenosis, choledochal cyst, and cholecystolithiasis, while the non-chronic biliary tract diseases included pancreatic duct stones and gallbladder polyps, and non-CCA cancers included pancreatic carcinoma (PC) and duodenal papilla carcinoma (Table 1). The included patients with CCA were mainly diagnosed by pathology from surgically resected specimens or biopsy specimens (open or laparoscopic surgical resection or ERCP-obtained biliary biopsy). Benign biliary diseases were diagnosed by imaging methods and laboratory tests and confirmed by endoscopy with a clinical follow-up of at least 1 year. During the 1-year clinical follow-up, special attention was paid to ensure that none of the patients with non-CCA diseases showed clinical or imaging signs of CCA. Patients with both CCA and bile duct stones were excluded.

**Table 1:** Patients characteristics

Characteristics	Cross-validation set		External validation set	
	**CCA**	**Non-CCA**	**CCA**	**Non-CCA**
Total number, *n* (%)	144 (38.3)	232 (61.7)	87 (33.6)	172 (66.4)
Age (years)				
Mean ± SD (range)	64.7 ± 9.3 (37–82)	60.8 ± 14.7 (18–89)	62.9 ± 10.4 (39–86)	59.3 ± 15.2 (22–85)
Gender, *n* (%)				
Male	92 (63.9)	101 (43.5)	53 (60.9)	99 (57.6)
Female	52 (36.1)	131 (56.5)	34 (39.1)	73 (42.4)
Tumor location, *n* (%)				
iCCA	6 (4.2)	–	4 (4.6)	–
pCCA	50 (34.7)	–	23 (26.4)	–
eCCA	88 (61.1)	–	60 (69.0)	–
TNM stage, *n* (%)				
I	25 (17.4)	–	9 (10.3)	–
II	63 (43.7)	–	16 (18.4)	–
III	22 (15.3)	–	29 (33.3)	–
IV	8 (5.6)	–	20 (23.0)	–
N	26 (18.0)	–	13 (14.9)	–
Non-CCA group, *n* (%)				
CBD	–	113 (48.7)	–	116 (67.4)
IBD	–	8 (3.4)	–	2 (1.2)
CBD and IBD	–	26 (11.2)	–	5 (2.9)
Pancreatic carcinoma	–	17 (7.3)	–	11 (6.4)
Pancreatic duct stones	–	14 (6.0)	–	8 (4.7)
Duodenal papilla carcinoma	–	4 (1.8)	–	2 (1.2)
Cholangitic stenosis	–	12 (5.2)	–	7 (4.1)
Cholecystolithiasis	–	26 (11.2)	–	15 (8.7)
Gallbladder polyps	–	6 (2.6)	–	3 (1.7)
Choledochal cyst	–	6 (2.6)	–	3 (1.7)
TBIL (μmol/L), *n* (%)				
≥ 23	128 (88.9)	131 (56.5)	72 (82.8)	101 (58.7)
< 23	16 (11.1)	101 (43.5)	15 (17.2)	71 (41.3)
GGT (U/L), *n* (%)				
≥ 60	136 (94.4)	166 (71.6)	82 (94.3)	110 (64.0)
< 60	8 (5.6)	66 (28.4)	5 (5.7)	62 (36.0)
TBA (μmol/L), *n* (%)				
≥ 10	114 (79.2)	96 (41.4)	62 (71.3)	69 (40.1)
< 10	30 (20.8)	136 (58.6)	25 (28.7)	103 (59.9)
CA19-9 (U/mL), *n* (%)				
≥ 34	117 (81.3)	73 (31.5)	75 (86.2)	64 (37.2)
< 34	27 (18.7)	159 (68.5)	12 (13.8)	108 (62.8)

*CCA* Cholangiocarcinoma, *N* TNM stage, *ERCP* biopsy with no TNM stage, *CBD* Common bile duct stones, *IBD* Intrahepatic bile duct stones, *TBIL* Total bilirubin, *GGT* Gamma-glutamyl transferase, *TBA* Total bile acid, *CA19-9* Carbohydrate antigen 19–9

In the external validation set, 259 patients were recruited from the Cancer Hospital of the Chinese Academy of Medical Sciences from January 2020 to May 2022, including 87 CCA patients (53 males and 34 females). There was no statistical difference in age between the CCA group and the non-CCA group (Table 1). Data of 63 features in the blood used for machine learning were collected from clinical information, including 37 blood biochemical features, 24 routine blood features, and two tumor biomarkers. General data of the 635 patients in the cross-validation set and external validation set was shown in Table 1, and the detailed information was listed in Additional file 2: Table S1.

### Clinical specimens

The bile collected from the bile duct was mainly obtained during ERCP, PTC, or surgery. None of the cancer patients received chemotherapy prior to cholangiography intervention or surgical treatment. Approximately 1 to 6 mL of bile (average 3 mL) was collected and transferred to a sterile tube each time. Bile and serum samples were shipped on ice immediately after being obtained, followed by centrifugation at 3000 × *g* for 15min at 4°C, and the supernatants were harvested and stored at − 80°C until the test.

### LC–MS/MS analysis and proteomic data analysis

Briefly, proteins in the bile and cell supernatant were extracted and quantified with the Brandford test, followed by alkylation and enzymatic digestion. The tryptic-digested peptides of the bile were then labeled by the iTRAQ reagent kit according to the manufacturer’s protocol but not with tryptic-digested peptides of cell supernatant. Then, the processed peptides of the bile and cell supernatant were performed by off-gel separation and nano-LC–MS/MS analysis. An EASY-Nlc 1000 nanoflow LC instrument coupled to a high-resolution mass spectrometer (Q Exactive Plus, Thermo Fisher Scientific) was used for LC–MS/MS analysis, the sample was injected into a tunnel-frit trap column and the trapped analytes were then separated by an analytical column, and the separated peptides were then identified and selected for data-dependent acquisition due to their electrical charge. After getting these experiment data, the raw files were analyzed by MaxQuant (version 1.6) and then were identified as corresponding proteins compared to those in the Swiss-Prot human protein sequence database (updated on 02/2019, 20,413 protein sequences). The false discovery rate of proteins was less than 1% (FDR < 1%) at both protein and peptide levels, and at least two peptides were identified for further data processing.

### Western blotting and quantitative real-time PCR (qRT-PCR)

The proteins in the bile were extracted by acetone precipitation. The protein solution was then separated by SDS/PAGE, transferred onto a PVDF membrane, and incubated overnight at 4°C with rabbit anti-CLU antibodies (1:1000, Cell Signaling) or mouse anti-GAPDH (1:2000, Proteintech). The membrane was later incubated with a secondary antibody of goat anti-mouse or anti-rabbit IgG (1:2000, Cell Signaling) and then visualized with chemiluminescence detection. RT-PCR was performed according to the instructions provided by the manufacturer using the qRT-PCR Kit (Thermo, USA). The forward and reverse PCR primers for CLU were 5′-GAGCAGCTGAACGAGCAGTTT-3′ and 5′-CTTCGCCTTGCGTGAGGT-3′ respectively; whereas for GAPDH, the forward primer was 5′-CCATCACCAT CTTCCAGG-3′, and reverse was 5′-ATGAGTCCTTCCACGATAC-3′. The relative expression levels of CLU mRNA were compared with GAPDH using the 2^−ΔΔCt^ value. Each experiment was repeated three times.

### Immunohistochemistry (IHC) staining

A cholangiocarcinoma TMA slide (containing 90 CCA tissues and 31 interlobular bile duct tissues) was purchased from Shanghai Outdo Biotech Co. Ltd. (Shanghai, China). Immunochemical Staining Kit (MXB, KIT-5002) and rabbit anti-CLU antibodies (1:400, Cell Signaling) were used for IHC staining. The images were analyzed by the Image pro plus 6.0 software. The expression intensity of CLU was judged by two senior pathologists independently without knowing any clinical and pathological data. Its staining intensity was divided into 4 grades: 0 represented negative expression (negative), 1 represented weak expression (weak), 2 was moderate expression (moderate), and 3 was strong expression (strong). Finally, negative, moderate, and weak expressions (0–2 points) were defined as low expression, and strong expression (3 points) was defined as high expression.

### Enzyme-linked immunosorbent assay-ELISA

The ELISA kits were used to detect the level of CLU (E-TSEL-H0014, Elabscience) and CA19-9 (E-EL-H0637c, Elabscience) in the bile or serum. First, the Reference Standard working solution and the samples are added to the Micro ELISA Plate, and then, the Biotinylated Detection Ab working solution is added immediately and incubated at 37°C for 90 min. Then aspirate or decant the solution from each well, add wash buffer to each well, and add HRP conjugate working solution to each well, then incubate for 30 min at 37°C. After washing again, add substrate reagent to each well and incubate for about 15–20 min at 37°C. Finally, add a Stop Solution to each well and determine the optical density (OD value) of each well at once with a micro-plate reader set to 450 nm. The bile and serum needed to be diluted before the test, and to measure the level of CLU, the bile and serum were diluted 100- and 5000-fold, respectively; to measure the level of CA199, the bile and serum were diluted 10,000- and fivefold, respectively.

### Deep learning method

Our deep learning method was mainly divided into three steps: feature selection, training for establishing the best diagnostic panel, and external validation. Firstly, a classification prediction model was built using the random forest (RF) method in the cross-validation set. Based on the tenfold cross-validation classification method, the data of 64 features from 376 patients were divided into ten parts, two of them were used for the testing cohort and eight of them were used for the training cohort. Then, the Least Absolute Shrinkage and Selection Operator (LASSO) method was used to select the best diagnostic panel based on the criteria that the prediction value of minimum top-ranking features number was the same with the entire features, and the prediction value was mainly evaluated by some popular metrics, such as AUC value, accuracy (ACC), specificity, and sensitivity. Finally, the deep learning panel was evaluated by an external validation set to obtain a robust classification performance. Random forest and LASSO were carried out in glmnet version 4.1–3.

### Statistical analysis

Continuous variables were expressed as median (interquartile range) or mean ± SD (standard deviation) and compared using the Mann–Whitney *U* test or Student’s *t*-test. Categorical variables were expressed as rate and compared with each other by the chi-square test. The expression of the same protein in different body fluids was compared by party rank sum test. Receiver operating characteristic (ROC) curves were used to evaluate the diagnostic performance of markers or panels and to establish cutoff levels, using the Youden index. The area under the curve (AUC) was calculated by the trapezoidal method, sensitivity, specificity, and accuracy (ACC) were calculated by standard 2 × 2 contingency tables, and a larger value represented better diagnostic performance. Decision curve analysis (DCA) was used to compare the diagnostic value of different clinical diagnostic models or markers. *t*-distributed stochastic neighbor embedding (tSNE) algorithm was used to visually evaluate the effects of the diagnostic model. *p*-values less than 0.05 were considered statistically significant. All analyses were performed with SPSS Statistics 20, GraphPad Prism version 7.0, and R version 4.1.0 (R Foundation for Statistical Computing; http://www.R-project.org).

## Results

### Proteomic profiles of bile and cell supernatant of CCA

**Fig. 1:**
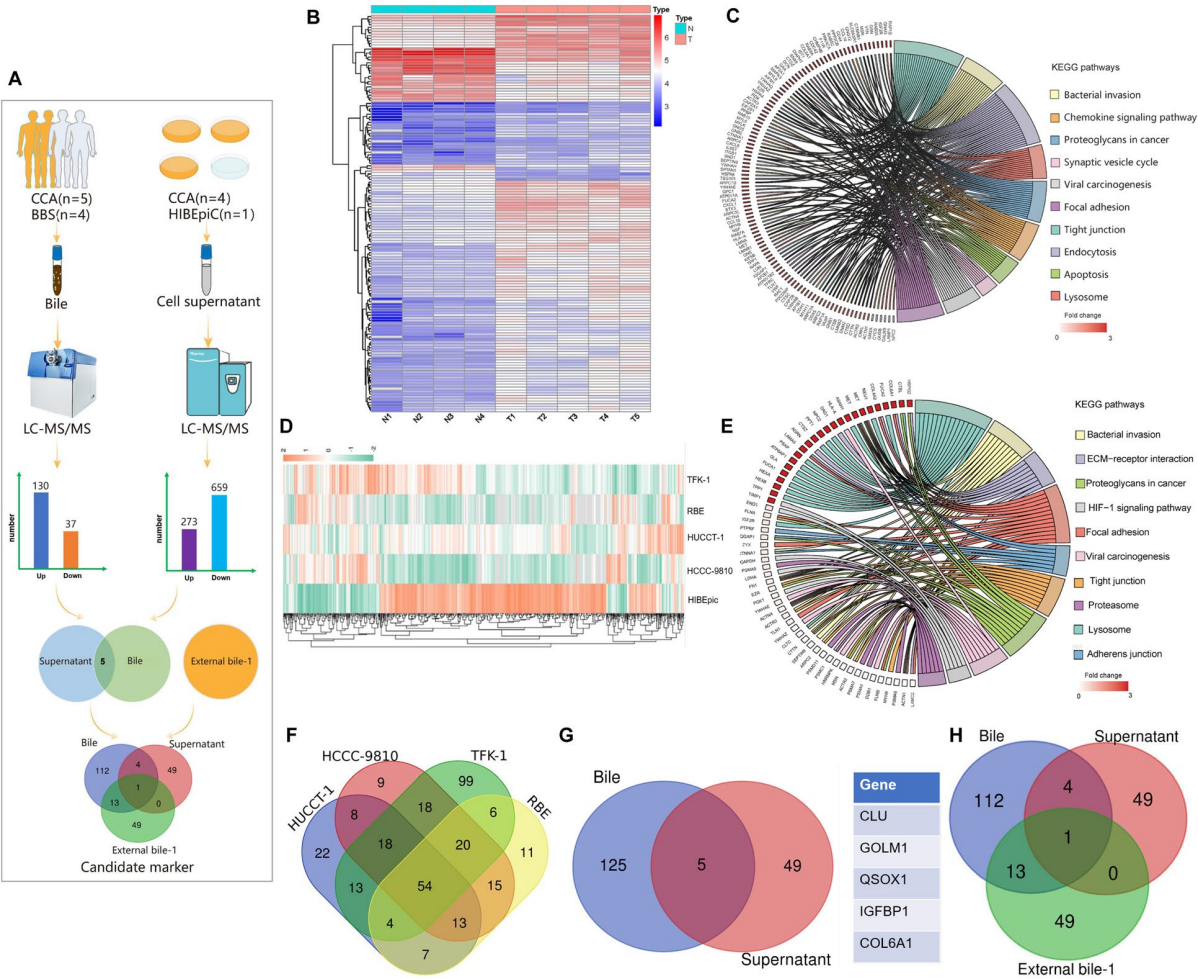
Identification of differentially expressed proteins in bile and cell supernatant. **A** The flow chart of screening CCA candidate markers by bile and cell supernatant proteomics. **B** The heatmap of differentially expressed proteins in bile of CCA and benign biliary diseases by using LC–MS/MS analysis; N1–N4 represented bile from four patients with benign biliary diseases, and T1–T5 represented bile from five CCA patients. **C** A chord dendrogram of the clustering of the differentially expressed proteins in bile by KEGG analysis. **D** The heatmap of differentially expressed proteins in cell supernatant. **E** A chord dendrogram of the differentially expressed proteins in supernatant by KEGG analysis. **F** A Venn diagram of 54 co-upregulated proteins in supernatant from four CCA cell lines by using LC–MS/MS analysis. **G** The five co-upregulated proteins both in bile and cell supernatant, their gene names were listed on the right. **H** A Venn diagram showing CLU protein was also upregulated in the proteomics data from another external bile group

Bile and cell supernatant proteomics were used to screen CCA candidate biomarkers (Fig. 1A). In the bile proteomics, 1585 proteins were identified, and 167 differentially expressed proteins were screened based on the standard of fold change ≥ 5.0 or ≤ 0.2 in comparison between CCA and benign biliary diseases, including 130 upregulated proteins and 37 downregulated proteins (Fig. 1A, B and Additional file 2: Table S2). The annotation analysis by Gene Ontology (GO) and Kyoto Encyclopedia of Genes and Genomes (KEGG) showed that these differentially expressed proteins were mainly related to tumorigenesis and cell-to-cell interactions, including chemokine signaling pathway, regulation of apoptotic signaling pathway, and endocytosis (Fig. 1C and Additional file 1: Fig. S2A).

Cell supernatant collected from four CCA cell lines (TFK-1, HuCCT-1, RBE, and HCCC-9810) and one human intrahepatic biliary epithelial cell line (HIBEpiC) were used for label-free quantitative analysis, and a total of 932 proteins differently expressed were found, including 273 upregulated proteins and 659 downregulated proteins (Fig. 1A, D and Additional file 2: Table S3). The GO and KEGG analysis indicated that these differentially expressed proteins were associated with signal transduction and immune regulation during tumor progression, including ECM-receptor interaction, endocytic vesicles, and endocytosis (Fig. 1E and Additional file 1: Fig. S2B).

There were 54 proteins elevated in CCA cell lines when compared with the HIBEpiC cell line (Fig. 1F). Five proteins were screened out when intersecting the upregulated proteins in the bile and supernatant (Fig. 1G), including CLU, COL6A1, GOLM1, QSOX1, and IGFBP1. Considering the limited number of our bile specimens, another bile proteomic dataset from the study by Marut Laohaviroj et al. (external bile 1) [13] was added. Based on the standard of fold change ≥ 1.5 in comparison between CCA and non-CCA, 63 upregulated proteins were identified in external bile 1, but only CLU was found elevated in external bile 1 among the five candidate proteins (Fig. 1H). Finally, CLU was selected for further studies.

### The overexpression of CLU in CCA

**Fig. 2:**
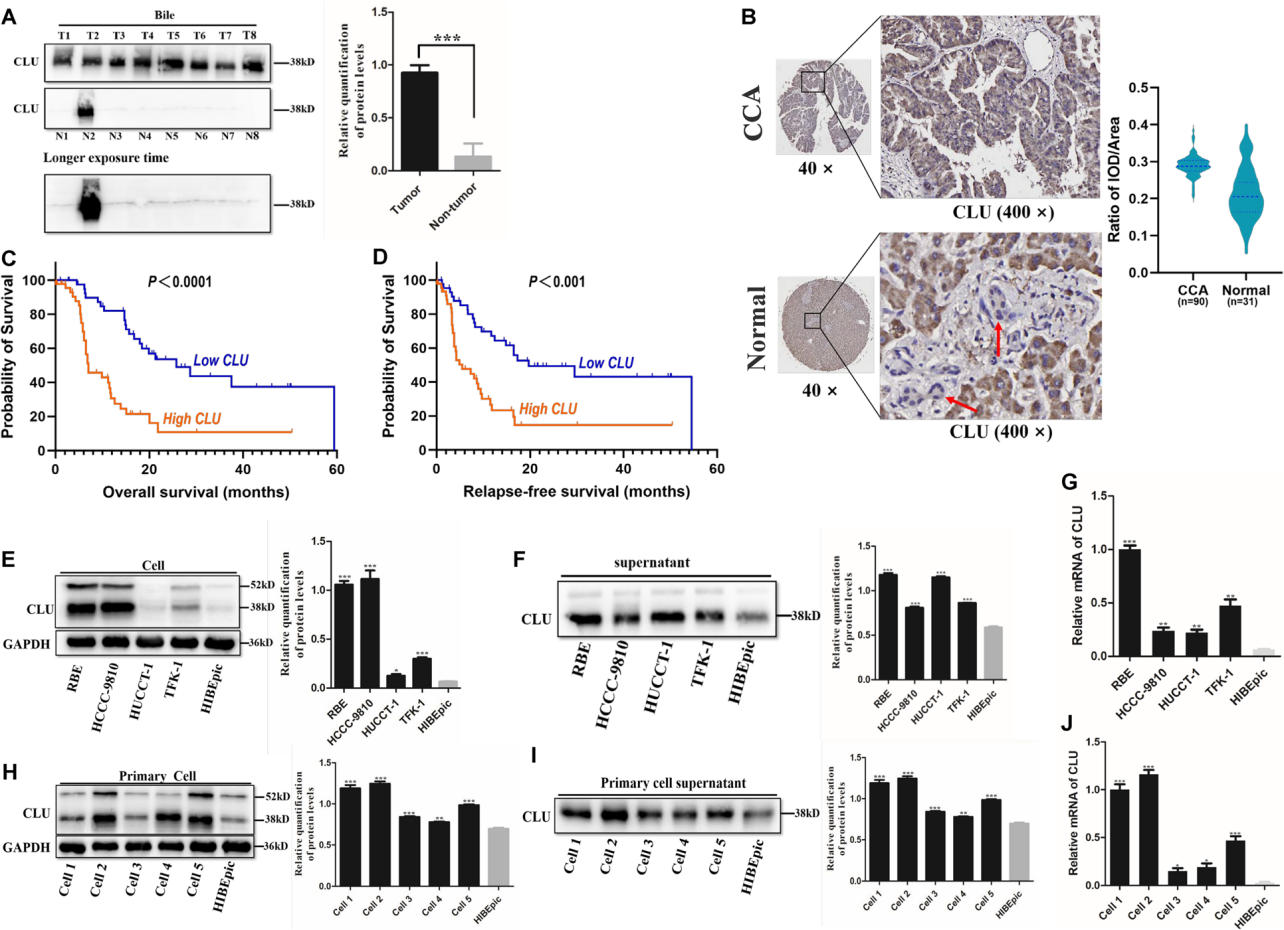
The overexpression of CLU in CCA. **A** Immunoblotting analysis of CLU in bile from 8 CCA patients and 8 benign biliary diseases patients. **B** Representative immunohistochemistry images and the level of CLU expression in CCA tissues and interlobular bile duct tissues (normal); the red arrow points to the interlobular bile duct. **C**, **D** The overall survival (OS) and relapse-free survival (RFS) curves of CLU in CCA; the blue represents low expression, and the orange represents high expression. **E**, **F** Immunoblotting analysis of CLU in cell and cell supernatant from four CCA cell lines and HIBEpiC cell line. **G** The mRNA level of CLU in four CCA cell lines and HIBEpiC cell line. **H**, **I** Immunoblotting analysis of CLU in cell and cell supernatant from five primary CCA cells and HIBEpiC cell. **J** The mRNA level of CLU in five primary CCA cells and HIBEpiC cell. *p < 0.05, **p < 0.01, ***p < 0.001

The level of CLU in CCA was verified in clinical specimens and cells. Sixteen bile samples (8 from CCA and 8 from benign biliary diseases) were collected for verifying the protein level of CLU. As shown in Fig. 2A, the level of CLU protein in CCA was higher, and there was little or no expression in the bile of benign biliary diseases. A tissue microarray (TMA) containing 121 surgical tissue specimens was used for immunohistochemistry staining, including 90 CCA tissues and 31 interlobular bile duct tissues (Additional file 1: Fig. S3). CLU was mainly located in the cytoplasm (Fig. 2B). Among the 90 CCA tissues, 89 were CLU-positive (98.9%). The positive tumor staining cases were then divided into weak, moderate, and strong expression, resulting in 4 (4.5%) cases, 36 (40.4%) cases, and 49 (55.1%) cases, respectively. In the 31 interlobular bile duct tissues, there were 12 (38.7%) negative staining cases. Analysis of immunohistochemistry images showed that the level of CLU in CCA was significantly higher (*p* < 0.001) (Fig. 2B). The Kaplan–Meier survival analysis indicated that CCA patients with high CLU level had shorter overall survival (OS) time (*p* < 0.0001) and shorter relapse-free survival (RFS) time (*p* < 0.001) (Fig. 2C, D). However, there was no association between CLU levels in CCA tumors and TNM stage, vascular invasion, lymph node affection, and metastasis (*p* > 0.05). Taken together, high expression of CLU could promote the progression of CCA.

As shown in Fig. 2E–G, the levels of CLU protein and mRNA were both highly expressed in four CCA cell lines (*p* < 0.05). We have successfully extracted five primary CCA cells from postoperative tissues, and CLU was also overexpressed in them (Fig. 2H–J).

### The diagnostic value of bile CLU and serum CA19-9 in CCA

**Fig. 3:**
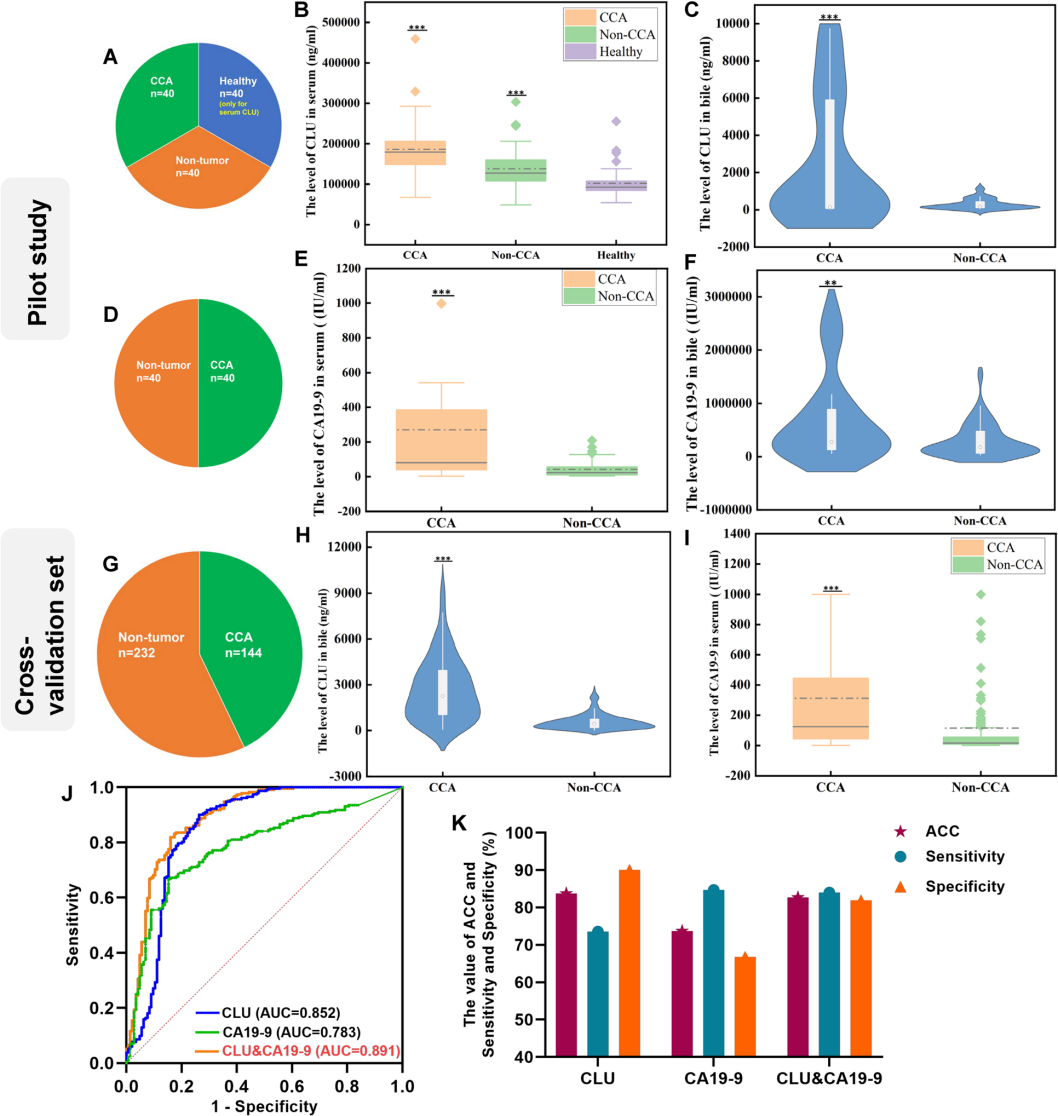
ELISA assay and ROC analysis of CLU or CA19-9 in the bile and serum. **A** A summary of the patient cohort used for pilot study of CLU. **B** ELISA analysis of CLU in serum from 40 CCA patients, 40 benign biliary diseases patients, and 40 healthy donors. **C** ELISA analysis of CLU in bile from 40 CCA patients and 40 benign biliary diseases patients. **D** A summary of the patient cohort used for the pilot study of CA19-9. **E**, **F** ELISA analysis of CA19-9 in the serum and bile from 40 CCA patients and 40 benign biliary diseases patients. **G** A summary of the patient cohort in cross-validation set. **H**, **I** ELISA analysis of bile CLU and serum CA19-9 from 144 CCA patients and 232 non-CCA patients. **J** The ROC curves of bile CLU, serum CA19-9, and CLU&CA19-9; the blue curve represents CLU, the green curve represents CA19-9, and the red curve represents CLU&CA19-9; the data means AUC (95%CI). **K** The accuracy (ACC), sensitivity, and specificity of bile CLU, serum CA19-9, and CLU&CA19-9. *p < 0.05, **p < 0.01, ***p < 0.001

To verify potential markers between bile CLU and serum CLU, 40 cases of bile and blood from patients with CCA or benign biliary diseases and 40 cases of blood from healthy donors were collected for pilot studies (Fig. 3A). As shown in Fig. 3B, C, the abundance of CLU in the serum and bile was higher in CCA when compared to the control group. The abundance of CLU in the serum was particularly high, with a median level even in healthy donors of 94,251.6 (83,887.5, 107,707.6) ng/mL. However, the median of bile CLU even in CCA was only 154.6 (73.7, 5945.9) ng/mL (Additional file 2: Table S4), significantly lower than levels in the blood (*p* < 0.001). High-abundant proteins are not suitable as diagnostic markers due to their low sensitivity [4]. Therefore, bile CLU was identified as a satisfactory diagnostic biomarker for CCA.

CA19-9 is found in both the serum and bile (Fig. 3D). As shown in Fig. 3E, F, the level of CA19-9 in the bile or serum was higher in CCA. In the CCA and benign biliary disease group, its median in bile was 280,276.9 (130,513.2, 906,704.6) IU/mL and 177,343.8 (65,142.4, 476,812.0) IU/mL, respectively, but in the serum, it was 81.1 (37.0, 389.0) IU/mL and 23.4 (9.0, 59.9) IU/mL, respectively (Additional file 2: Table S4), significantly lower than levels in bile (*p* < 0.001). In the same way, CA19-9 in the serum was more suitable as a diagnostic biomarker for CCA.

After that, a total of 376 patients were brought into the cross-validation set for further study (Fig. 3G). As shown in Fig. 3H, J, the abundance of bile CLU in CCA was higher, and ROC analysis showed a satisfactory diagnostic capacity with an AUC of 0.852 (95%CI 0.806 to 0.899) (sensitivity of 73.6%, specificity of 90.1%). Bile CLU in CCA was significantly higher than in benign biliary diseases and non-CCA cancers, and there was also no statistical difference in bile CLU concentration between malignant controls and non-malignant controls (*p* = 0.23) (Additional file 1: Fig. S4A), and there was no correlation between bile CLU level and age (*p* = 0.60) or tumor stages in patients with CCA (*r*_s_ =  − 0.104, *p* = 0.260) (Additional file 1: Fig. S4B). Comparing bile CLU levels between patients with chronic biliary tract diseases and those with non-chronic biliary tract diseases, no statistical difference was found between them (Additional file 1: Fig. S4C). In addition, there was no difference in bile CLU level between lithiasis-associated CCA patients (*n* = 20) and single CCA patients (*p* = 0.26), but a significant difference was found between lithiasis-associated CCA patients and cholangiolithiasis patients (*p* < 0.001)( Additional file 1: Fig. S4D). The above results indicated that there was no association between bile CLU concentrations and chronic biliary tract conditions.

The abundance of serum CA19-9 was higher in CCA, and its AUC value was 0.783 (95%CI 0.735 to 0.830) (sensitivity of 84.7%, specificity of 66.8%) (Fig. 3I, J). Due to the fact that bile CLU had a high specificity and a low sensitivity, while CA19-9 was just the opposite, we consider establishing a model containing bile CLU and serum CA19-9 for better accuracy. As shown in Fig. 3J, the diagnostic value increased significantly in the CLU&CA19-9 model with an AUC of 0.891 (95%CI 0.855 to 0.928), much higher than its two individual members. Its sensitivity and specificity were both improved to 84.0% and 81.9%, respectively (Fig. 3K) (Additional file 3: Table S5), indicating a better diagnostic performance.

### Biomarker panel development by deep learning

**Fig. 4:**
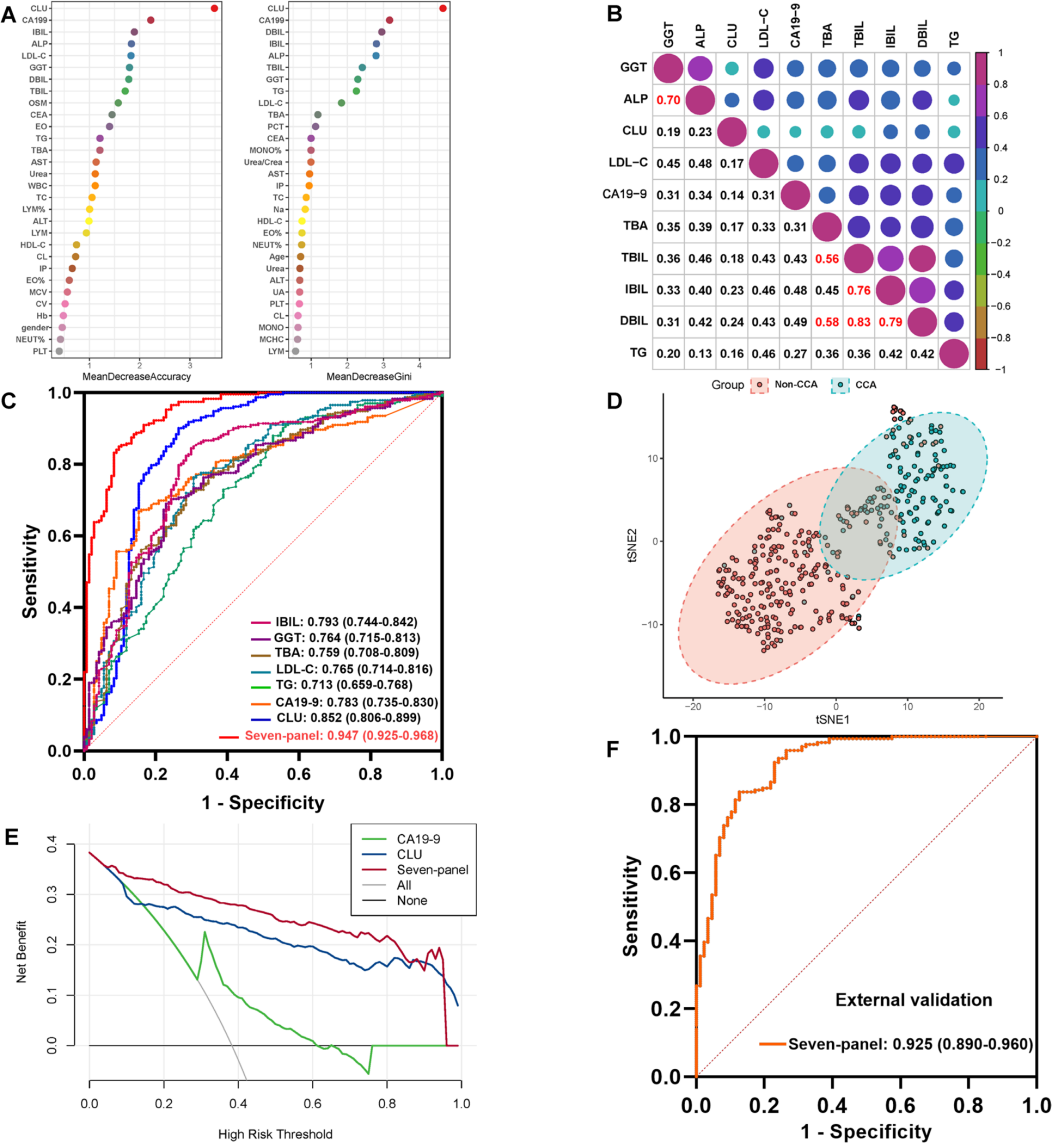
The diagnostic panel development by machine learning. **A** The 30 top-ranked features were screened by random forest model, and they were ranked by accuracy (left) and Gini index (right); the features closer to the upper right were more important. **B** The correlation matrix between the top 10 features, including CLU, CA19-9, DBIL, IBIL, ALP, TBIL, GGT, TG, LDLC, and TBA; the numbers represent the correlation coefficient (*r*) between the two features. **C** ROC curves of the seven-panel and its members; the data means AUC (95%CI). **D** tSNE analysis of the seven-panel; the blue represents CCA, and the pink represents non-tumor. **E** The DCA analysis of the seven-panel, CLU and CA19-9; the green represents CA19-9, blue represents CLU, and red represents the seven-panel. **F** ROC curve of the seven-panel in the external validation set; the data means AUC (95%CI). AUC is the area under the curve. *r* ≥ 0.8 represents high correlation, 0.5 ≤ *r* < 0.8 represents strong correlation, 0.3 ≤ *r* < 0.5 represents weak correlation, and *r* < 0.3 represents no correlation

The diagnostic performance of a biomarker can be improved by combining it with different types of circulating biomarkers. Data of each patient used for machine learning contained bile CLU and 63 serum features. In the cross-validation set, the top 30 features were screened out according to their accuracy (the left) and Gini index (the right) by random forest (RF) (Fig. 4A), and CLU, CA19-9, and bilirubin contributed most to the RF model. Their diagnostic values were identified by ROC analysis (Additional file 3: Table S6), and only the top 10 features had satisfactory AUC values, including CLU, CA19-9, DBIL, IBIL, ALP, TBIL, GGT, TG, LDLC, and TBA. A low correlation between different biomarkers could enhance the content of the message and improve the diagnostic performance [14]. The correlation matrix between the 10 markers showed that there was a high correlation between TBIL, DBIL, and IBIL (*r* > 0.5, *p* < 0.001), which could be explained by their clinical relationship, similar to the same principle applied to the high correlation between GGT and ALP (*r* > 0.5, *p* < 0.001) (Fig. 4B). The other markers had low correlations with each other (*p* < 0.05).

An optimal selection about the number of features is critical in establishing a classification model. Based on the top 10 selected markers, the LASSO method was applied to establish an optimal classification model (Additional file 1: Fig. S5A). Finally, the seven-panel was identified to be the optimal diagnostic model, including CLU, CA19-9, IBIL, GGT, TG, LDLC, and TBA, and there was little or no correlation (*r* < 0.5) between the seven biomarkers (Fig. 4B). As shown in Fig. 4C, the seven-panel model had a much higher AUC value (AUC: 0.947, 95%CI: 0.925 to 0.968), and its sensitivity and specificity were both improved to 90.3% and 84.9%, indicating a great diagnostic accuracy (ACC of 87.0%) (Additional file 3: Table S5), and the seven-panel model had no correlation with TNM stage (*p* = 0.410), lymph node metastasis (*p* = 0.537), and distant metastasis (*p* = 0.537).

tSNE algorithm was used to simplify the complex confusion matrix, which can help us visualize and intuitively understand the distribution of diseases. The tSNE result of the seven-panel showed that the CCA group and control group formed different clusters (Fig. 4D), indicating that even under visualization conditions, the seven-panel could also distinguish CCA well. Decision curve analysis (DCA) was used to observe the clinical performance of CLU, CA19-9, and the seven-panel, and the result showed that the seven-panel boosted more clinical overall benefits in differentiating CCA (Fig. 4E).

### External validation for the seven-panel model

In order to further evaluate the stability and reliability of the seven-panel model, we applied it to an independent external validation set of 259 patients, including 87 patients with CCA and 172 patients with non-CCA diseases (Table 1). In the external validation set, the seven-panel performed a satisfactory prediction ability with an AUC of 0.925 (Fig. 4F), achieving a sensitivity of 87.4% and specificity of 83.7% (Additional file 3: Table S5). The tSNE and DCA analysis also showed a perfect diagnostic power (Additional file 1: Fig. S5B and 5C). In summary, these results suggested that the seven-panel could accurately diagnose CCA and meet the actual needs of clinical decision-making.

### Web server of CCA diagnosis according to the panel

**Fig. 5:**
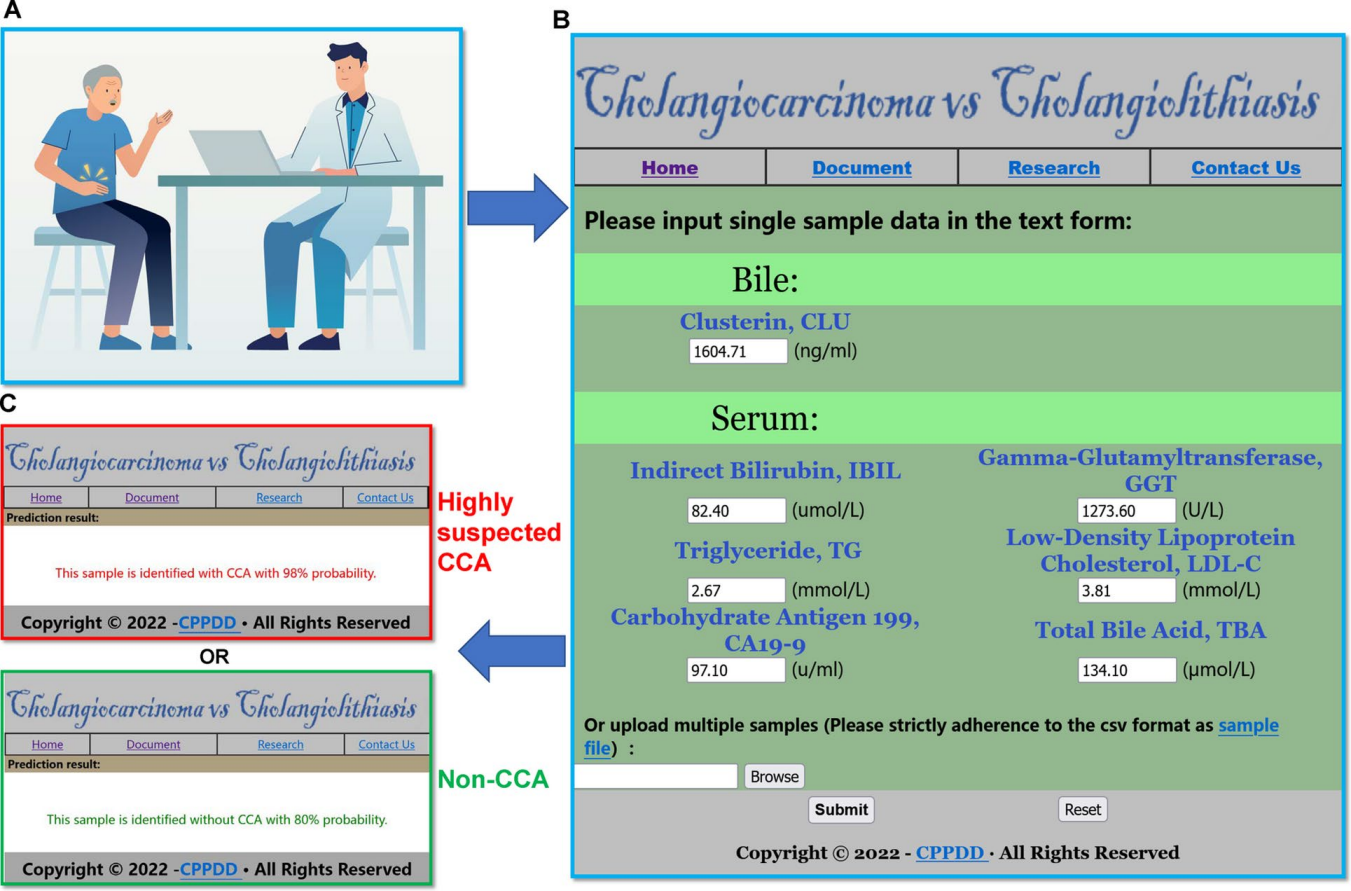
The diagnostic website of CCA. **A** Patients with biliary tract diseases seeking medical help. **B** Simple web interface to input the value of the seven markers. **C** The result page of the online website; the risk of CCA was expressed as percentages probabilities

To facilitate the use of this model in clinical practice, we established a user-friendly online model on the China Prediction Platform of Digital Disease (CPPDD) (available at: http://cppdd.cn/CCA/). Users need only input the specific values of the seven biomarkers, and then click the “Submit” button (Fig. 5). After calculation, the model would show a conclusion that whether this patient has CCA or not with a percentage probability. Users should double-check the units to ensure correct results.

## Discussion

**Fig. 6:**
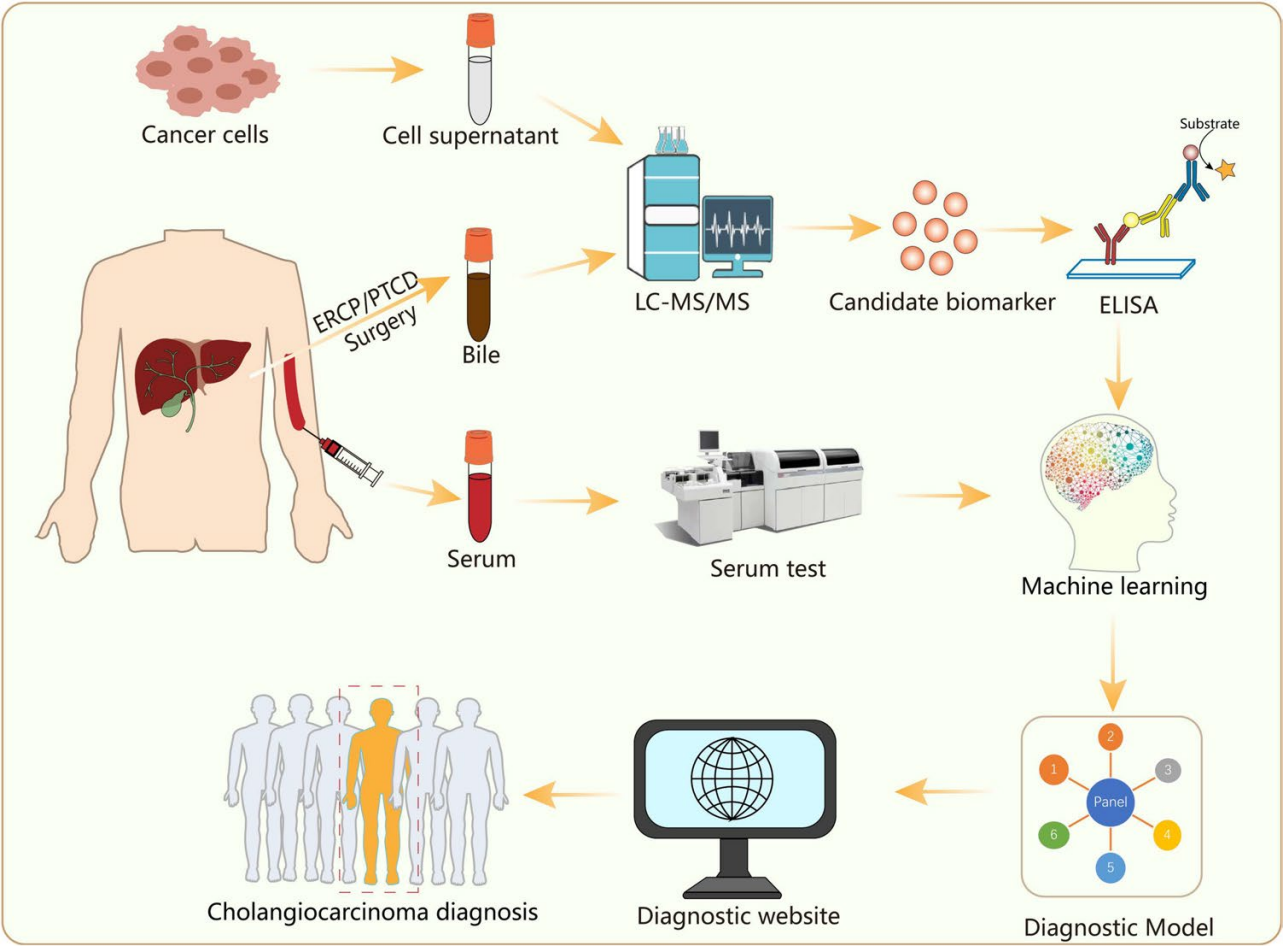
The overall workflow of establishing a seven-panel model and an online prediction platform for CCA

In this study, we innovatively discovered a novel diagnostic biomarker CLU for CCA by proteomics. To improve its diagnostic accuracy, deep learning algorithms were used to develop a diagnostic model comprised of bile and serum biomarkers (Fig. 6). Until now, this is the largest and most comprehensive study combining bile and serum biomarkers to differentiate CCA.

Accurately diagnosing CCA has always been a huge pain, and searching for new diagnostic methods is urgent. The high heterogeneity of CCA can lead to inaccurate results from primary tumor-based proteomic, but body fluids reflecting global changes in pathophysiological status are a great source for searching novel and reliable diagnostic markers [15]. Moreover, proteins in bile are abundant and relatively easy to detect, so identifying the differently expressed proteins in bile turned out to be a good strategy for searching novel diagnostic biomarkers.

During the discovery stage, a total of 1585 proteins were identified in bile proteomic, much higher than previous studies [16–18]. These differently expressed proteins were mainly associated with tumorigenesis, indicating specificity for CCA. In order to confirm that the differential proteins in bile were indeed secreted by tumor cells rather than inflammatory cells or other cells, we performed proteomics of supernatant from CCA cells and HIBEpiC, and considering the limitations of single-center studies, we have cited bile proteomics data from another study [13]. Finally, the analysis of three proteomic data showed that protein CLU was the most suitable biomarker for CCA.

Clusterin (CLU) is a stress-activated, ATP-independent molecular chaperone, normally secreted from cells, and can promote cancer development by regulating cell proliferation, apoptosis, and cycle. It is highly expressed in several cancers, including esophagus, prostate, and breast [19, 20]. Although Li et al. performed the immunohistochemistry of CLU in 13 cases of CCA tissues, they did not conduct further studies [21], and this study was the first to deeply and comprehensively explore its diagnostic ability in CCA. In this study, the overexpression of CLU in CCA was identified in cell lines, primary cells, and clinical specimens. We also found a close correlation between CLU and CCA prognosis. Currently, the most commonly used diagnostic marker for cholangiocarcinoma in clinical practice is CA19-9, although its diagnostic effect is moderate [22].

Previous studies have pointed out that serum CLU can be used for cancer diagnosis [23, 24]. Before verifying the diagnostic utility of CLU for CCA, pilot studies were performed to identify its expression level in different types of specimens. The results showed that CLU in the serum was particularly noteworthy, while its abundance in bile was relatively low. The main probable reason is that in addition to being secreted in cholangiocytes, CLU is also secreted into the serum from other organs, such as the brain, heart, liver, lung, and prostate [25, 26]. However, high-abundance proteins are less sensitive for diagnosis, and low-abundance proteins are just the opposite [4]. Therefore, bile CLU was selected as a potential diagnostic marker for CCA. CLU is a secreted protein that can be secreted by tissues into body fluids [27], and this study indicates that CLU is overexpressed in CCA tissues; in addition, bile is the body fluid adjacent to CCA tissues, so the elevated bile CLU concentrations in CCA patients may be secreted from the cancer tissues. Several studies using proteomics found a number of differentially expressed proteins specific for CCA in the bile, such as Mac-2BP, SSP411, and AAT, but due to their small validation cohort size (26 to 54 CCA subjects) and only utilizing a single marker, their diagnostic ability was limited [13, 28, 29]. In our study, a total of 644 subjects were enrolled to identify the diagnostic value of bile CLU and serum CA19-9, and ROC analysis showed that bile CLU was significantly superior to the diagnostic capacity of CA19.9 (DeLong test, *p* < 0.05). CLU had a good specificity but lacked sensitivity, while CA19-9 was just the opposite due to its high expression in some benign biliary diseases. But combining them into a panel improved the diagnostic value for CCA, and there were 33 CCA patients with Lewis-antigen negative (CA19-9 < 40 IU/mL), of which 81.8% (27 patients) had elevated bile fluid CLU. In conclusion, the diagnostic value of two mutually compensating indicators can be significantly improved by combining them.

During the development of CCA, various indicators in the blood changed, such as transaminases and bilirubin, but they lack CCA specificity and are also similarly changed in other biliary diseases [30]. Each biomarker in the blood and bile reflects the unique characteristics of the patient, and we suspected that combining several different types of biomarkers may result in a better diagnostic tool. Deep learning is always used to learn logical patterns by analyzing mass data with mathematical algorithms and to make prediction models. In recent years, deep learning has been widely used in cancer diagnosis and prognosis prediction models and has been identified to improve the accuracy of cancer recurrence and survival prediction [31]. In this work, deep learning was used to build a diagnostic model consisting of bile CLU and other serum markers.

To establish a multi-markers diagnostic panel, each marker should have diagnostic power and should not correlate with each other, so as to make sure that each marker possesses unique information about the stage of the patient [32]. Based on the above criteria, a seven-panel model was established from 64 markers in the cross-validation set by random forest and the LASSO method. Compared with its individual components, the diagnostic capacity of the generated model was significantly improved (DeLong test, *p* < 0.05), and when compared to the combination of CLU and CA19-9, the seven-panel had a better prediction power with AUC increasing from 0.891 to 0.947 (Additional file 3: Table S5). As the best dimension reduction visualization available at this time, tSNE can help us visualize and intuitively understand the distribution of the disease [14]. The tSNE algorithm showed that the seven-panel could visually distinguish CCA effectively, indicating that the tSNE algorithm can be applied to the visualization output of the diagnostic model of CCA. The prediction power of the seven-panel model was then validated in an external validation set, which was completely independent from the modeling process.

The prediction model contained seven markers, including bile CLU, CA19-9, indirect bilirubin (IBIL), gamma-glutamyl transferase (GGT), low-density lipoprotein cholesterol (LDL-C), triglyceride (TG), and total bile acid (TBA). Serum bilirubin mainly includes direct bilirubin (DBIL), indirect bilirubin (IBIL), and total bilirubin (TBIL), and they are often elevated due to liver function impairment or biliary obstruction [33]. Gamma-glutamyl transferase (GGT) is an enzyme of glutathione and cysteine metabolism and is the standard liver enzyme test reflecting cholestasis and bile duct obstruction [34]. Total bile acids (TBA) are the end product of cholesterol metabolism in the liver, and it always increases during hepatocellular lesions or biliary obstruction [35]. In summary, GGT, IBIL, and TBA reflected biliary strictures, and they always increase during CCA development [36, 37]. LDL-C was mainly related with cardiovascular diseases, but in a prospective cohort study, LDL-C levels were found to be closely associated with cancer mortality, which may be due to cholesterol levels [38]. Cholesterol and its metabolites play an important role in tumor biology, especially in oncogenic signaling pathways, ferroptosis, and tumor microenvironment [39]. Lipid accumulation can aggravate tumor progression via AMP-kinase and mTOR signaling, and TG plays an irreplaceable role in this pathway [40, 41]. In summary, the seven biomarkers are closely related to cancer development.

There are many methods to distinguish CCA from benign biliary strictures, but their results are frustrating (Additional file 3: Table S7). Endoscopic retrograde cholangiopancreatography (ERCP) fluoroscopy with brush cytology (ERCP-BC) (and/or forceps biopsy) or fine needle aspiration (FNA) is the primary sampling technique for identifying CCA, but their poor predictive values (pooled sensitivity of 6–65%) are often challenged by insufficient amount of tissue specimens and location and size of the lesion [5, 42]. Serum proteins were also used for CCA diagnosis, such as fucosylated fetuin-A, CA50, and MMP-7 (pooled sensitivity of 55.3–75%, specificity of 78–90%) [43–45]. The extracellular vesicles in the bile and serum also were used for CCA diagnosis albeit with poor AUC value (0.696–0.759) [46, 47]. Lately, proteins and nucleic acids in bile were used for CCA diagnosis as well, such as decreased total bile acids, protein CMA1, and MCM-5, but their sensitivity or specificity was low [48, 49]. In the non-invasive methods, proteins and exosomes in urine also could be used to distinguish CCA with an AUC value of 0.68–0.82 [50, 51]. In contrast, our method could distinguish CCA better, providing a diagnostic option for patients who might not be keen on surgery, while also adding to the current CCA evaluation tool available.

CCA is a rapidly developing tumor and the technique of bile collection is highly demanding, and many studies only managed to enroll a small cohort size, while we collected a large number of bile samples from two centers. In addition, this is the first study to use deep learning to combine bile and serum markers for CCA diagnosis. Finally, for further application, a user-friendly prediction platform for CCA was established online.

There were also some limitations in our study. This study population is entirely Chinese, and a larger cohort study involving patients with CCA or benign biliary diseases from multiple medical centers will be conducted to fully verify the diagnostic ability of this diagnostic model and strive for early application in clinical diagnosis. Another limitation is that it is difficult to collect bile because ERCP and other related surgical procedures are not available in all medical facilities, but fortunately, these techniques have become more widely known in recent years. In addition, the oncogenic mechanism of CLU in CCA has not been elucidated; thus, cell and animal experiments are needed to explore it in the later stage.

## Conclusions

To the best of our knowledge, this is the largest and most comprehensive study using different body fluid biomarkers to efficiently distinguish CCA. This study established a multi-markers diagnostic panel for CCA utilizing a discovery-verification-validation pipeline. Our findings suggest that the seven-panel model would be a promising method to predict the occurrence of CCA.

### Supplementary Information

**Additional file 1:** **Fig. S1.** Study design. **Fig. S2.** The Biological Process (BP), Cellular Component (CC) and Molecular Function (MF) of clustering of the differentially expressed proteins in bile (A) and supernatant (B) by GO analysis. **Fig. S3.** The immunohistochemistry images of tissue microarray (TMA). **Fig. S4.** (A) The expression level of bile CLU in CCA, non-CCA cancers and benign biliary diseases. (B) The expression level of bile CLU at different TNM stages in CCA patients. (C) The expression level of bile CLU in each benign biliary disease. (D) The expression level of bile CLU in lithiasis-associated CCA group, single CCA group and lithiasis group. **Fig. S5.** (A) Lasso Cox regression analysis of 10 candidate markers. (B) and (C). tSNE and DCA analysis in external validation set.

**Additional file 2: Table S1. **The detailed information of the 635 patients in cross-validation set and external validation set.** Table S2. **1585 proteins were identified in the bile proteomics.** Table S3. **932 proteins differently expressed were found in cell supernatant proteomics.** Table S4. **The abundance of CLU and CA19-9 in serum or bile in pilot studies.

**Additional file 3:** **Table S5.** The diagnostic values of biomarkers and panels. **Table S6.** The AUC values of the top 30 features. **Table S7.** Other methods for CCA diagnosis.

## Data Availability

The mass spectrometry proteomics data have been submitted to the ProteomeXchange Consortium via the iProX partner repository with the dataset identifier PXD043745.

## Abbreviations

ACC: Accuracy
AUC: Area under the curve
CA19-9: Carbohydrate antigen 19–9
CBD: Common bile duct
CCA: Cholangiocarcinoma
CLU: Clusterin
DBIL: Direct bilirubin
DCA: Decision curve analysis
ERCP: Endoscopic retrograde cholangiopancreatography
GGT: Gamma-glutamyl transferase
IBD: Intrahepatic bile duct
IBIL: Indirect bilirubin
LDL-C: Low-density lipoprotein cholesterol
PTC: Percutaneous transhepatic cholangiography
RF: Random forest
TBA: Total bile acid
TBIL: Total bilirubin
TG: Triglyceride
TMA: Tissue microarray
