# Correction: Identification of a novel bile marker clusterin and a public online prediction platform based on deep learning for cholangiocarcinoma

**DOI:** 10.1186/s12916-024-03404-0

**Published:** 2024-04-29

**Authors:** Long Gao, Yanyan Lin, Ping Yue, Shuyan Li, Yong Zhang, Ningning Mi, Mingzhen Bai, Wenkang Fu, Zhili Xia, Ningzu Jiang, Jie Cao, Man Yang, Yanni Ma, Fanxiang Zhang, Chao Zhang, Joseph W. Leung, Shun He, Jinqiu Yuan, Wenbo Meng, Xun Li

**Affiliations:** 1 The First School of Clinical Medicine, Lanzhou University, Lanzhou, 730030 Gansu China; 2 Department of General Surgery, The First Hospital of Lanzhou University, Lanzhou, 730030 Gansu China; 3 Gansu Province Key Laboratory of Biological Therapy and Regenerative Medicine Transformation, Lanzhou, 730030 Gansu China; 4 School of Medical Information and Engineering, Xuzhou Medical University, Xuzhou, 221004 Jiangsu China; 5 Clinical Research Center, Big Data Center, The Seventh Affiliated Hospital, Sun Yat-Sen University, Shenzhen, 518107 Guangdong China; 6 Division of Gastroenterology, UC Davis Medical Center and Sacramento VA Medical Center, Sacramento, CA 95817 USA; 7 Department of Endoscopy, National Cancer Center/National Clinical Research Center for Cancer/Cancer Hospital, Chinese Academy of Medical Sciences and Peking Union Medical College, Beijing, China

**Correction** **:** **BMC Med 21, 294 (2023)**

**https://doi.org/10.1186/s12916-023-02990-9**

Following the publication of the original article it was brought to our attention that the published version of the manuscript retained reference to “deep learning” where Deep learning methods where initially employed in the study following subsequent rounds of revision these techniques were removed from the study. A combination of machine learning and other statistical methodologies replaced the deep learning techniques and as such this term should be replaced throughout the manuscript.
