# Supplementary material for: Identification of a novel bile marker clusterin and a public online prediction platform based on deep learning for cholangiocarcinoma
Source: BMC Med. 2023 Aug 8;21:294. doi: 10.1186/s12916-023-02990-9 (PMC10408060; doi:10.1186/s12916-023-02990-9)
Supplement: Supplementary file 1 — Additional file 1: Fig. S1. Study design. Fig. S2. The Biological Process (BP), Cellular Component (CC) and Molecular Function (MF) of clustering of the differentially expressed proteins in bile (A) and supernatant (B) by GO analysis. Fig. S3. The immunohistochemistry images of tissue microarray (TMA). Fig. S4. (A) The expression level of bile CLU in CCA, non-CCA cancers and benign biliary diseases. (B) The expression level of bile CLU at different TNM stages in CCA patients. (C) The expression level of bile CLU in each benign biliary disease. (D) The expression level of bile CLU in lithiasis-associated CCA group, single CCA group and lithiasis group. Fig. S5. (A) Lasso Cox regression analysis of 10 candidate markers. (B) and (C). tSNE and DCA analysis in external validation set. [file 12916_2023_2990_MOESM1_ESM.docx]

**Identification of a novel bile marker Clusterin and a public online prediction platform based on machine learning algorithm for Cholangiocarcinoma**

Long Gao^#1,2^ Yanyan Lin^#1,2^ Ping Yue^#1,2^ Shuyan Li^#3^ Yong Zhang^1,2^ Ningning Mi^1,2^ Mingzhen Bai^1,2^ Wenkang Fu^1,2^ Zhili Xia^1,2^ Ningzu Jiang^1,2^ Jie Cao^1^ Man Yang^4^ Yanni Ma^1^ Fanxiang Zhang^1^ Joseph W. Leung^5^ Shun He^6*^ Jinqiu Yuan^4*^ Wenbo Meng^1,2*^ Xun Li^1,2^


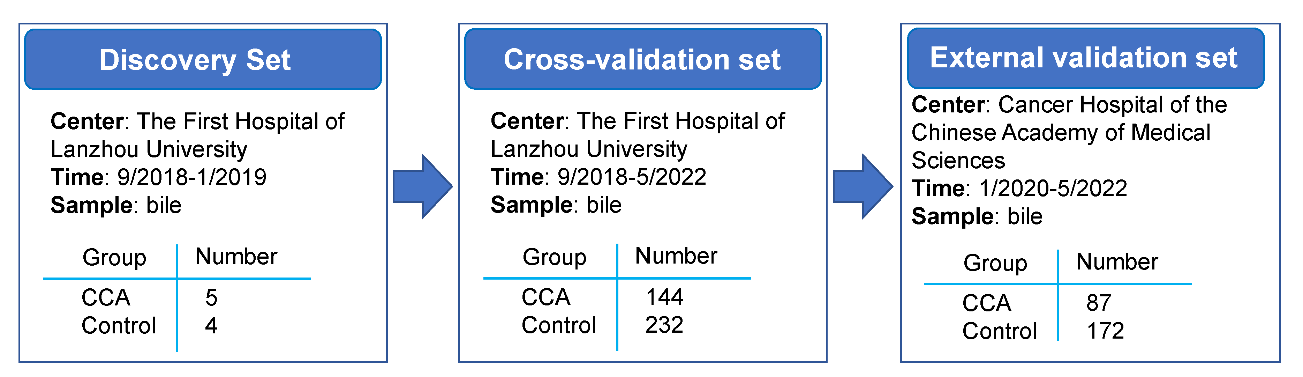
**Figure S1** Study design. Discovery set was used for bile proteomics, Cross-validation set was used to establish a diagnostic model by machine learning, External validation set was an independent cohort for validating the diagnostic value of the model.


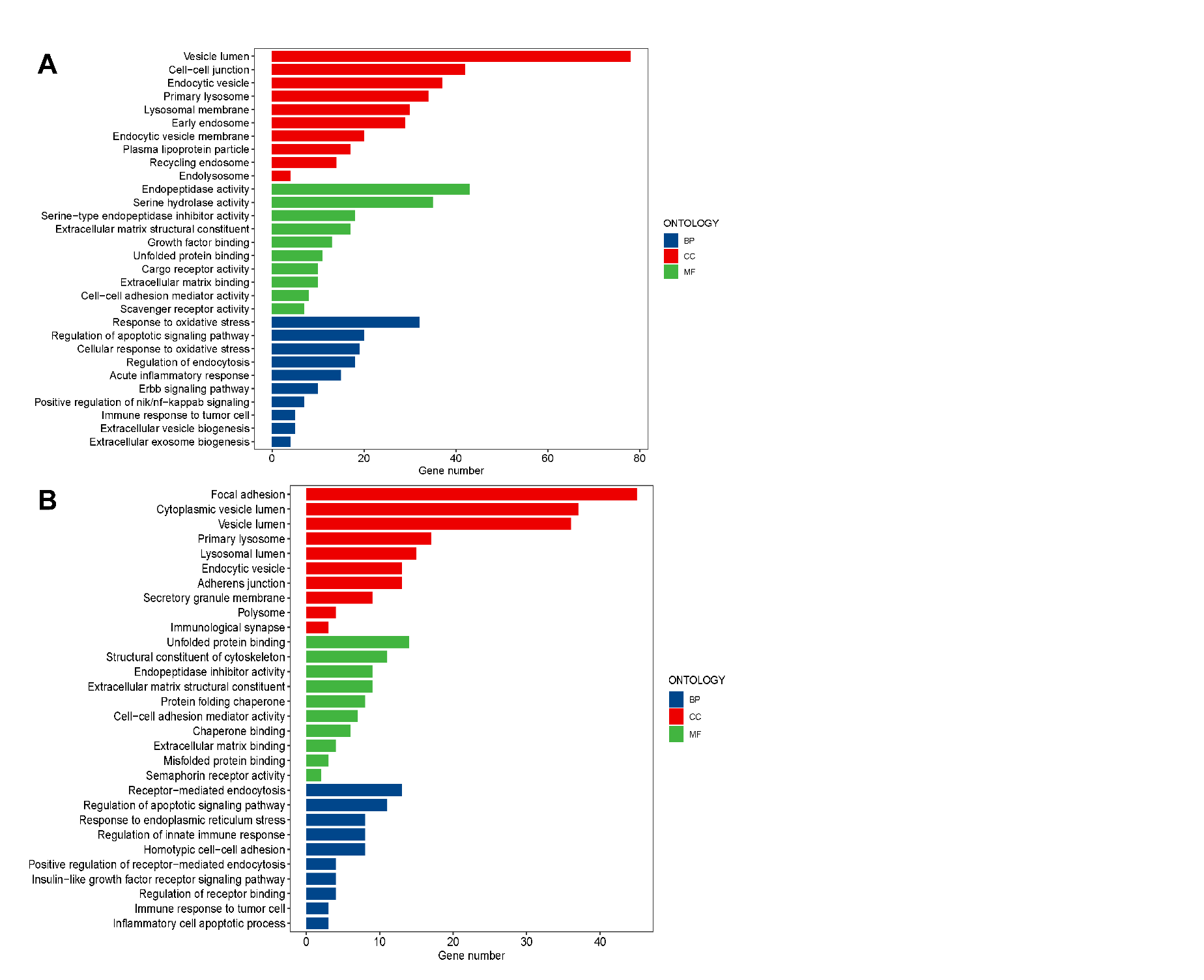
 **Figure S2 A** and **B** The Biological Process (BP), Cellular Component (CC) and Molecular Function (MF) of clustering of the differentially expressed proteins in bile and supernatant by GO analysis.

**Figure S3** The immunohistochemistry images of tissue microarray (TMA), each circle represents a tissue of CCA or interlobular bile duct **
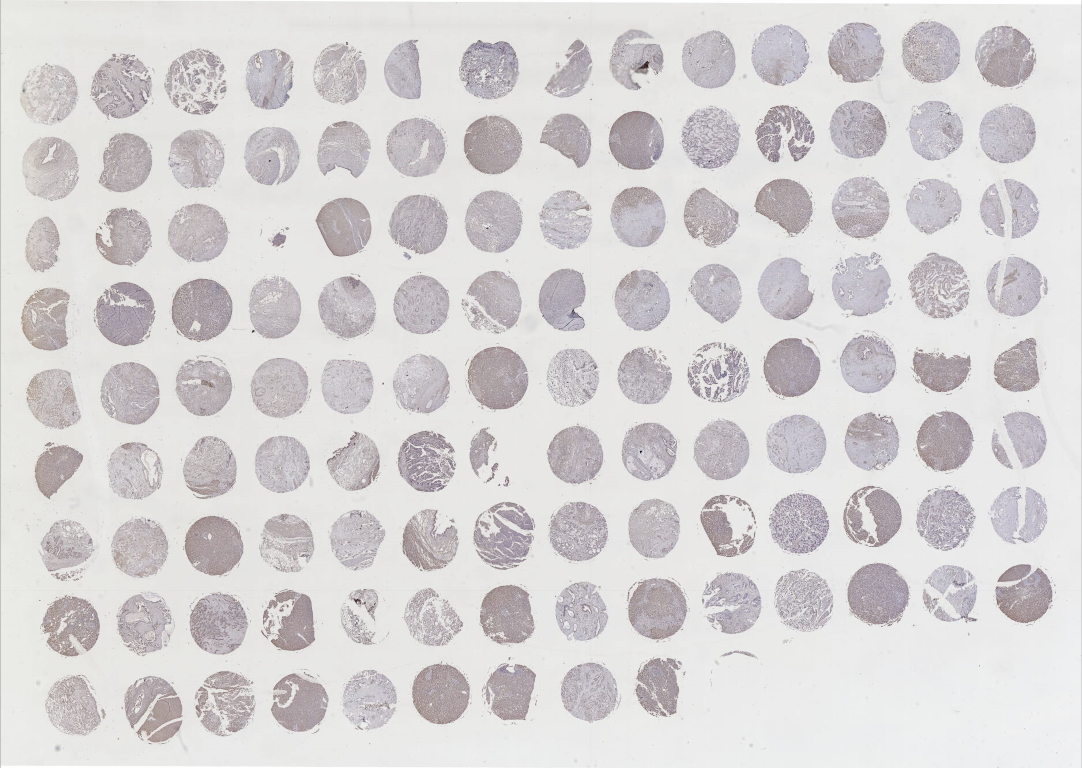
**


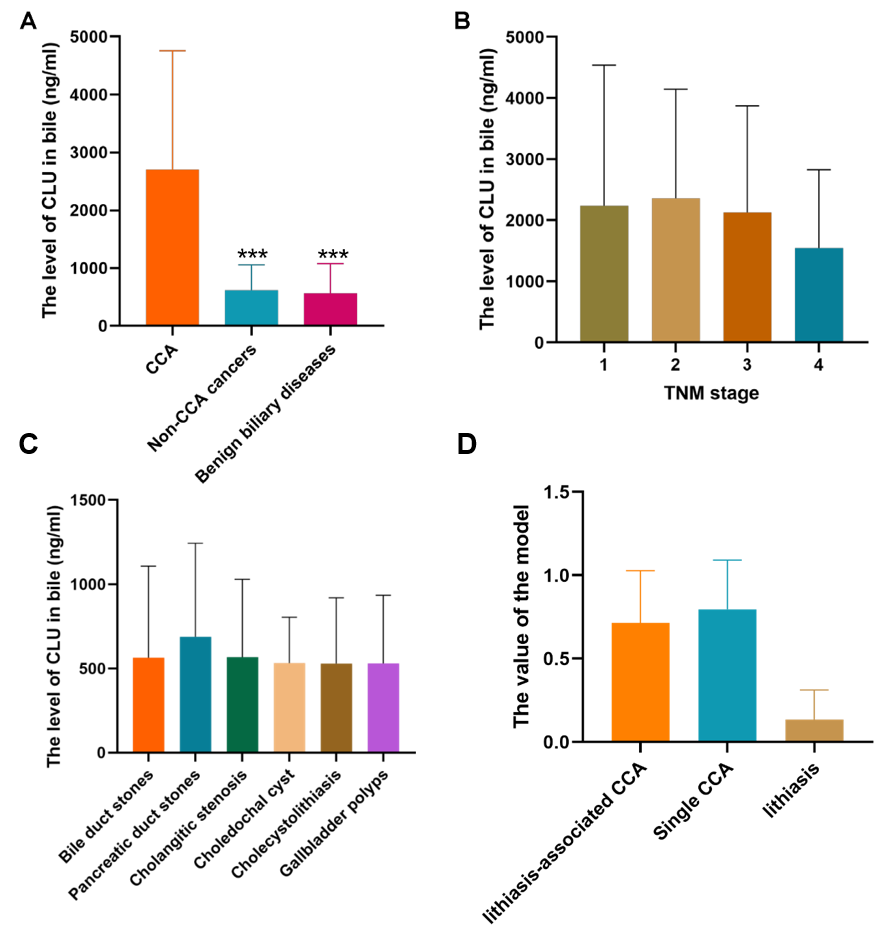


**Figure S4 A** The expression level of bile CLU in CCA, non-CCA cancers and benign biliary diseases. **B** The expression level of bile CLU at different TNM stages in CCA patients. **C** The expression level of bile CLU in each benign biliary disease. **D** The expression level of bile CLU in lithiasis-associated CCA group, single CCA group and lithiasis group. *P ＜0.05, **P＜0.01, ***P＜0.001.


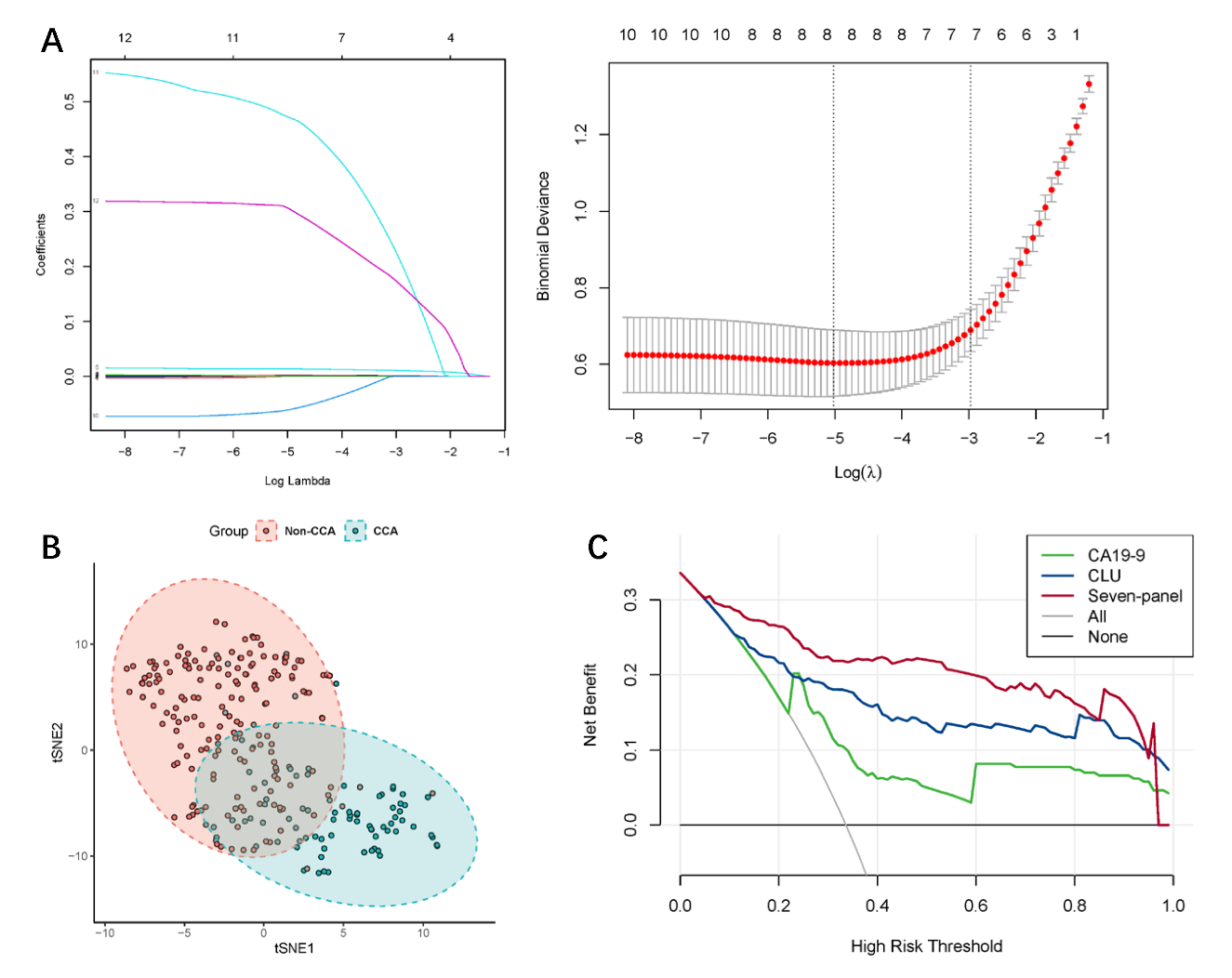
 **Figure S5** **A** Lasso Cox regression analysis of 10 candidate markers. **B** and **C** tSNE and DCA analysis in external validation set.
