# Supplementary material for: Identification of a novel bile marker clusterin and a public online prediction platform based on deep learning for cholangiocarcinoma
Source: BMC Med. 2023 Aug 8;21:294. doi: 10.1186/s12916-023-02990-9 (PMC10408060; doi:10.1186/s12916-023-02990-9)
Supplement: Supplementary file 3 — Additional file 3: Table S5. The diagnostic values of biomarkers and panels. Table S6. The AUC values of the top 30 features. Table S7. Other methods for CCA diagnosis. [file 12916_2023_2990_MOESM3_ESM.docx]

**Identification of a novel bile marker Clusterin and a public online prediction platform based on machine learning algorithm for Cholangiocarcinoma**

Long Gao^#1,2^ Yanyan Lin^#1,2^ Ping Yue^#1,2^ Shuyan Li^#3^ Yong Zhang^1,2^ Ningning Mi^1,2^ Mingzhen Bai^1,2^ Wenkang Fu^1,2^ Zhili Xia^1,2^ Ningzu Jiang^1,2^ Jie Cao^1^ Man Yang^4^ Yanni Ma^1^ Fanxiang Zhang^1^ Joseph W. Leung^5^ Shun He^6*^ Jinqiu Yuan^4*^ Wenbo Meng^1,2*^ Xun Li^1,2^

**Table S5** The diagnostic values of biomarkers and panels

| **Name** | **Sensitivity** | **Specificity** | **ACC** | **AUC** |
| --- | --- | --- | --- | --- |
| CLU | 73.6% | 90.1% | 83.8% | 0.852 |
| CA19-9 | 84.7% | 66.8% | 73.7% | 0.783 |
| TG | 61.1% | 72.8% | 68.4%% | 0.713 |
| LDL-C | 69.4% | 76.3% | 73.7% | 0.765 |
| TBA | 70.1% | 72.4% | 71.5% | 0.759 |
| GGT | 77.1% | 69.0% | 72.1% | 0.764 |
| IBIL | 70.1% | 84.5% | 79.0% | 0.793 |
| CLU & CA19-9 | 84.0% | 81.9% | 82.7% | 0.891 |
| Seven-panel (Cross-validation) | 90.3% | 84.9% | 87.0% | 0.947 |
| Seven-panel (External validation) | 87.4% | 83.7% | 84.9% | 0.925 |

ACC: accuracy; AUC: area under the curve

**Table S6** The AUC values of the top 30 features

| **Name (Abbreviation)** | **AUC** | **95% CI** | ***P*** |
| --- | --- | --- | --- |
| Clusterin (CLU) | 0.852 | 0.806-0.899 | <0.001 |
| Carbohydrate antigen (CA19-9) | 0.783 | 0.735-0.830 | <0.001 |
| Direct bilirubin (DBIL) | 0.806 | 0.759-0.853 | <0.001 |
| Indirect bilirubin (IBIL) | 0.793 | 0.753-0.849 | <0.001 |
| Alkaline phosphatase (ALP) | 0.774 | 0.725-0.822 | <0.001 |
| Total bilirubin (TBIL) | 0.801 | 0.753-0.849 | <0.001 |
| Gamma-glutamyl transpeptidase (GGT) | 0.764 | 0.715-0.813 | <0.001 |
| Triglyceride (TG) | 0.713 | 0.659-0.768 | <0.001 |
| Low-density lipoprotein cholesterol (LDL-C) | 0.765 | 0.714-0.816 | <0.001 |
| Total Bile Acid (TBA) | 0.759 | 0.708-0.809 | <0.001 |
| Procalcitonin (PCT) | 0.663 | 0.607-0.719 | <0.001 |
| Carcinoembryonic antigen (CEA) | 0.647 | 0.590-0.703 | <0.001 |
| Monocyte %(MONO%) | 0.616 | 0.559-0.674 | <0.001 |
| Urea/Crea | 0.600 | 0.541-0.658 | 0.001 |
| Aspartate Transaminase (AST) | 0.695 | 0.641-0.748 | <0.001 |
| Inorganic Phosphorus (IP) | 0.502 | 0.441-0.562 | 0.958 |
| Total Cholesterol (TC) | 0.673 | 0.615-0.731 | <0.001 |
| Na | 0.644 | 0.587-0.702 | <0.001 |
| High-density lipoprotein cholesterol (HDL-C) | 0.669 | 0.613-0.726 | <0.001 |
| CL | 0.680 | 0.626-0.734 | <0.001 |
| Osmotic pressure (OSM) | 0.656 | 0.599-0.712 | <0.001 |
| Eosinophils (EO) | 0.639 | 0.582-0.696 | <0.001 |
| Urea | 0.620 | 0.562-0.679 | <0.001 |
| Alanine transaminase (ALT) | 0.685 | 0.632-0.739 | <0.001 |
| Eosinophils % (EO%) | 0.599 | 0.540-0.648 | 0.001 |
| White blood cell (WBC) | 0.577 | 0.519-0.635 | 0.012 |
| Lymphocyte (LYM) | 0.547 | 0.488-0.605 | 0.128 |
| Mean Corpuscular Volume (MCV) | 0.525 | 0.465-0.585 | 0.420 |
| Lymphocyte % (LYM%) | 0.545 | 0.486-0.603 | 0.145 |
| Neutrophil% (NEUT%) | 0.515 | 0.456-0.573 | 0.635 |

| Method | Sample number | Sample | Sensitivity | Specificity | AUC |
| --- | --- | --- | --- | --- | --- |
| This method | 635 | Bile/Serum | 90.3% | 84.9% | 0.95 |
| ERCP-BC | 60 | Tissue | 6-64% | 98-100% |  |
| ERCP-FNA | 26-226 | Tissue | 45-65% | 100% |  |
| Serum proteins | 78-108 | Serum | 55.3-75% | 78-90% | 0.64-0.81 |
| Serum EVs | 73 | Serum | 69.8-88.4% | 63.3-70% | 0.76-0.70 |
| Bile acids | 49 | Bile | 88.9% | 87.1% |  |
| Bile proteins | 97 | Bile | 65.4% | 78% | 0.79 |
| Bile EVs | 96 | Bile | 67% | 97% |  |
| Urinary proteins | 87 | Urine | 71% | 72% | 0.82 |
| Urinary EVs | 43 | Urine | 63.6% | 66.7-71.4% | 0.68 |

**Table S7** Other methods for CCA diagnosis
